# The Temozolomide Mutational Signature: Mechanisms, Clinical Implications, and Therapeutic Opportunities in Primary Brain Tumor Management

**DOI:** 10.3390/cells15010057

**Published:** 2025-12-29

**Authors:** Adar Yaacov, Roni Gillis, Jaber Salim, Daniela Katz, Noam Asna, Iddo Paldor, Albert Grinshpun

**Affiliations:** 1 Helmsley Cancer Center, Shaare Zedek Medical Center, Jerusalem 9103102, Israel; 2 Faculty of Medicine, The Hebrew University of Jerusalem, Jerusalem 7610001, Israel; 3 Neurosurgery Department, Shaare Zedek Medical Center, Jerusalem 9103102, Israel

**Keywords:** glioma, Temozolomide, mutational signatures, personalized medicine

## Abstract

Temozolomide (TMZ) remains foundational in the management of adult-type diffuse gliomas in general, and glioblastoma specifically. However, its efficacy harbors an evolutionary trade-off. TMZ drives its cytotoxicity through generating O^6^-methylguanine lesions, especially active in MGMT-silenced, mismatch repair (MMR)-proficient tumors. By selecting for acquired MMR-deficient subclones, often via MSH6 inactivation, this process escalates into a hypermutator phenotype, generating thousands of de novo alterations. This is a hallmark of the mutational signature known as SBS11, characterized by C>T transitions, which is associated with TMZ treatment. The hypermutator phenotype drives heterogeneity, therapeutic resistance, spatial diversification, and distant recurrence. Despite harboring a mutational burden comparable to melanoma and lung cancer, TMZ-induced hypermutation does not sensitize gliomas to immune checkpoint blockade. This resistance reflects the profoundly immunosuppressive brain microenvironment, impaired antigen presentation, marked transcriptional plasticity, and perhaps also the frequent use of corticosteroids. Emerging strategies aim to exploit vulnerabilities created by TMZ-mediated genomic instability, including PARP, ATR, WEE1, and AURKA inhibition; alternative alkylators; metabolic rewiring; and G-quadruplex stabilization. Notably, the real-time detection of evolving mutational signatures via CSF-based liquid biopsies may enable adaptive therapy before radiographic progression. By reframing TMZ as a potent evolutionary agent rather than a conventional chemotherapy, this review synthesizes recent mechanistic insights and translational opportunities to guide a next-generation, evolution-informed treatment paradigm for glioma.

## 1. Introduction

Adult-type diffuse gliomas are primary brain tumors, typically molecularly classified into three subtypes: glioblastoma, IDH-wildtype (CNS WHO grade 4); oligodendroglioma, IDH-mutant, and chromosome 1p/19q co-deletion (CNS WHO grades 2–3); and astrocytoma, IDH-mutant (CNS WHO grades 2–4); each of which can be further classified into molecular subtypes [1]. Gliomas are associated with high morbidity and mortality rates, with glioblastoma standing as the most aggressive and lethal primary brain tumor in adults, with a dismal median overall survival (OS) of approximately 15 months despite maximal therapeutic intervention [1,2,3,4,5]. The current standard of care (SoC) for glioblastoma and high-risk IDH-mutant gliomas, established by the landmark Stupp article, combines maximal safe surgical resection with concurrent radiotherapy and temozolomide (TMZ) chemotherapy, followed by adjuvant TMZ maintenance therapy [1,5,6]. TMZ, an orally bioavailable alkylating agent with excellent central nervous system penetration, became the cornerstone of first-line chemotherapy for glioblastomas and progressive lower-grade gliomas. O^6^-methylguanine-DNA methyltransferase (MGMT) promoter methylation, present in approximately 45% of IDH-wildtype glioblastomas and 50–70% of IDH-mutant gliomas, emerged as the key predictive biomarker for treatment response [7,8,9,10,11,12]. Yet this therapeutic success harbored an evolutionary reckoning. The paradigm shift began with the recognition that TMZ is not merely a cytotoxic agent but a potent mutagen that fundamentally reshapes tumor evolutionary trajectories [13,14]. Whole-genome sequencing (WGS) revealed an elevated tumor mutational burden (TMB) after TMZ treatment, defined by a distinctive mutational footprint, or mutational signature SBS11, characterized by C>T transitions at GpC dinucleotides [15,16,17]. This mutagenesis process has profound implications for tumor evolution, therapy resistance, and, in theory, potentially increasing neoantigen production and immunotherapy response [18,19,20].

In this review, we provide an overview of the mechanistic understanding of TMZ-induced mutagenesis, dissect the clinical implications of the hypermutator phenotype with a particular focus on immunotherapy consequences, and propose therapeutic opportunities arising from treatment-driven evolution. We examine biomarkers for predicting and monitoring hypermutation, evaluate emerging DNA damage response (DDR)-targeted and immunotherapy strategies, and explore evolutionary-informed treatment paradigms. By reframing TMZ from a standard chemotherapy to a driver of tumor evolution, evolution-aware management of brain malignancies might be beneficial.

## 2. Molecular Mechanisms of the TMZ Signature

### 2.1. The Mechanistic Core: DNA Alkylation, MGMT, and Mismatch Repair

TMZ is an oral alkylating agent of the imidazotetrazine class and exerts its cytotoxic effects through DNA alkylation, generating multiple adducts that include N^7^-methylguanine (70%), N^3^-methyladenine (9%), and the highly mutagenic O^6^-methylguanine (O^6^-meG, 5%) [21]. TMZ is a prodrug that spontaneously converts at physiological pH to its active metabolite. It has excellent oral bioavailability and blood–brain barrier penetration, critical advantages for treating central nervous system (CNS) malignancies. While numerically minor, O^6^-meG lesions drive TMZ’s therapeutic efficacy and mutagenic potential; in the absence of repair, O^6^-meG mispairs with thymine during DNA replication, initiating futile cycles of mismatch repair (MMR)-mediated excision and re-synthesis [21]. These lesions, if unrepaired, lead to futile cycles of MMR and ultimately cell death—the mechanism underlying TMZ’s therapeutic benefit [19,21,22].

**Figure 1:**
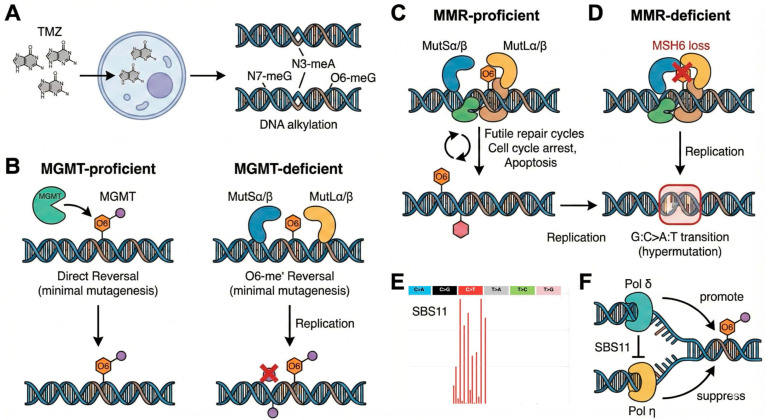
Mechanistic model of TMZ-induced mutagenesis and the SBS11 signature. (**A**) Temozolomide (TMZ) enters the cell and alkylates DNA, producing N^7^-methylguanine (70%), N^3^-methyladenine (9%), and the highly mutagenic O^6^-methylguanine (O^6^-meG, 5%). (**B**) In MGMT-proficient cells, the MGMT enzyme directly removes the methyl group from the O^6^ position, restoring the guanine. In MGMT-deficient cells, the O^6^-meG lesion persists. (**C**) In the presence of functional mismatch repair (MMR), the complex recognizes the O^6^-meG:T mispair formed during replication. This initiates futile repair cycles that lead to DNA double-strand breaks, cell cycle arrest, and apoptosis (the intended therapeutic effect). (**D**) In the absence of functional MMR (e.g., via MSH6 loss), the O^6^-meG:T mispair escapes recognition. A second round of replication fixes the mispair into a G:C>A:T transition mutation. (**E**) This process generates the characteristic SBS11 mutational signature, defined by C>T transitions. (**F**) DNA polymerase δ plays a key role in generating SBS11 mutations, while DNA polymerase η acts to suppress this signature during trans-lesion synthesis. Google’s Nano Banana Pro assisted in creating the illustration.

The MGMT enzyme directly reverses O^6^-meG lesions in a stoichiometric reaction (transferring the methyl group to a cysteine residue, thereby inactivating itself). Thus, MGMT promoter methylation silences this repair pathway and strongly predicts TMZ response [7,8,9,10,11]. However, the MGMT-MMR axis creates a potential double-edged sword: patients whose tumors respond to TMZ (MGMT-methylated, MMR-proficient) face the highest risk of therapy-induced hypermutation if MMR function is subsequently lost [20] (Figure 1A–D).

### 2.2. The SBS11 Mutational Signature

Initial studies using WGS of paired primary and corresponding recurrent gliomas exposed a recurrent mutational pattern: the molecular footprint of TMZ exposure was captured in mutational signature SBS11, characterized by C>T transitions enriched at GpC dinucleotides—the molecular fingerprint of unrepaired O^6^-meG lesions [16,17]. This results in a hypermutation phenotype, defined by over 10 mutations per megabase (Mut/Mb) and often exceeding 100 Mut/Mb (Figure 1D,E). Hypermutation affects 30–57% of TMZ-treated IDH-mutant low-grade gliomas at recurrence and is associated with accelerated progression, distant recurrence patterns, and shortened survival [20,23,24]. Comparable data in IDH-wildtype glioblastoma remain limited, as most patients succumb to disease before hypermutation-driven recurrence can be fully characterized, though the phenomenon has been documented in recurrent IDH-wildtype glioblastoma [24].

While the evidence that TMZ exposure can result in the development of hypermutator phenotypes is fairly robust [13,20], the direct attribution to the SBS11 signature has faced scrutiny [15,25]. A notable 2019 study by Kucab et al. found that TMZ exposure in human-induced pluripotent stem cells resulted in predominantly T>C substitutions. Conversely, the signature of treatment with 1,2-Dimethylhydrazine, a potent alkylating agent with known carcinogenicity, was found to be very similar to SBS11. Nonetheless, recent 2025 studies reconciled this finding and showed in nearly 40 lymphoblast cell lines that an SBS11-like pattern was induced by TMZ upon sequential inactivation of DNA repair pathways. MMR deficiency resulted in TMZ resistance, allowing for the accumulation of ultra-hypermutator phenotypes with correspondence to SBS11 [26]. Mechanistically, Sanyal et al. have biochemically demonstrated the mechanistic basis of SBS11 formation [17]. In vitro studies using purified DNA polymerases and TMZ-damaged templates revealed that DNA polymerase δ (Pol δ), the major replicative polymerase, efficiently generates SBS11-like mutation spectra when encountering O^6^-meG lesions. The mutation pattern arises from template-strand mispairing: O^6^-meG preferentially pairs with thymine rather than cytosine, resulting in G:C>A:T transitions upon replication. The characteristic CpG enrichment reflects the methylation specificity of TMZ at guanine residues within CG dinucleotides. Lastly, Chowdhury et al. resolved the initial conflict in a longitudinal analysis of the Glioma Longitudinal Analysis (GLASS) consortium cohort and found two distinct TMZ-associated mutational signatures: the classic SBS11, and a T>C dominated signature, SBS119, thereby reconciling the preclinical finding by Kucab et al. from 2019 [27].

Interestingly, Sanyal et al. also found that human DNA polymerase η (Pol η) suppresses SBS11-like mutations, suggesting that trans-lesion synthesis polymerases (a damage-tolerance mechanism that allows replication machinery to bypass bulky DNA lesions) may modulate the mutagenic consequences of TMZ therapy [17] (Figure 1F). These findings, subject to replication and validation studies, have potential translational implications: enhancing Pol η activity might reduce hypermutation risk, while Pol η inhibition could paradoxically enhance TMZ cytotoxicity at the cost of increased mutagenic burden.

### 2.3. MMR Deficiency: Amplifier of the Hypermutator Phenotype

The transition from moderate mutation accumulation to frank hypermutation occurs through acquired MMR deficiency, most frequently via MMR gene mutations (up to 91% of TMZ-treated hypermutator phenotypes), mainly biallelic inactivation or mutations in the MSH6 gene (more than 40%), including a recurrent p.T1219I alteration [20,27,28,29]. Multi-region sequencing and phylogenetic reconstruction studies reveal that MMR-deficient subclones emerge under TMZ selection pressure, expanding clonally to dominate recurrent tumors [14,20]. The hypermutator phenotype is defined not merely by mutation (which can reach up to ultra-hypermutator > 200 Mut/Mb) but by the overwhelming enrichment of SBS11 and related signatures (SBS119) indicative of TMZ mutagenesis; recent analyses from the GLASS consortium, encompassing 206 IDH-mutant gliomas with paired primary and recurrent samples, demonstrated enrichment for SBS11 and SBS119 signatures after TMZ treatment in both astrocytoma and oligodendroglioma subtypes [14,27]. In addition, hypermutation was identified in 30–57% of recurrent low-grade gliomas previously exposed to TMZ, indicating that this is not a rare phenomenon but rather a common evolutionary trajectory [20,23].

Post-transcriptional regulation adds complexity to MMR function in gliomas. The RNA-binding protein MEX3A, upregulated following TMZ exposure, binds MSH2 mRNA and promotes its degradation, effectively inducing functional MMR deficiency [30]. This epigenetic mechanism provides an additional pathway to hypermutation beyond genetic MSH6 inactivation.

Overall, TMZ-induced mutagenesis, characterized by SBS11, can be vastly elevated with concurrent MMR deficiency. Its evolutionary consequences are an active field of research.

## 3. Tumor Evolution Under TMZ Pressure

### 3.1. Evolutionary Trajectories: Clonal Dynamics and Malignant Transformation

**Figure 2:**
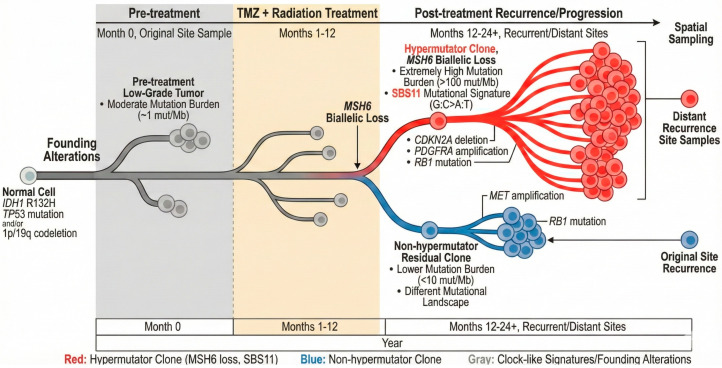
Phylogenetic evolution of gliomas under TMZ selection pressure. The diagram traces the evolutionary history of a glioma from a normal cell through treatment to recurrence. Pre-treatment: the founding clone (typically a low-grade tumor) carries initial alterations (e.g., *IDH1* mutation, 1p/19q co-deletion) and a moderate mutational burden (~1 Mut/Mb). Treatment: Exposure to TMZ and radiation creates a bottleneck and selective pressure. Hypermutator recurrence (red branch): acquisition of *MSH6* loss leads to a hypermutator phenotype. This lineage undergoes rapid clonal expansion characterized by the SBS11 signature and an extremely high mutational burden (>100 Mut/Mb). These clones frequently acquire additional drivers (e.g., *CDKN2A* deletion, *RB1* mutation) and are often found at distant recurrence sites. Non-hypermutator recurrence (blue branch): alternatively, a residual clone retaining MMR proficiency may drive recurrence. These tumors maintain a lower mutational burden (<10 Mut/Mb) and follow a distinct evolutionary trajectory (e.g., *MET* amplification), typically recurring at the original tumor site. Google’s Nano Banana Pro assisted in creating the illustration.

TMZ treatment fundamentally reshapes glioma evolutionary landscapes, driving divergent trajectories [14,31]. In recurrent astrocytoma, hypermutation is associated with malignant transformation into grade 4 disease and the acquisition of additional oncogenic drivers. Hypermutation was statistically significantly associated with high-grade disease at recurrence with an odds ratio of 12 in a patient cohort of n = 82 and with significantly worse prognoses compared to recurrent tumors without hypermutation, with a hazard ratio of 3.4 [23]. Phylogenetic reconstruction using WGS illuminates the temporal emergence of hypermutator clones [31]. Early truncal alterations (IDH1/2 mutations, TP53 mutations in astrocytomas, 1p/19q co-deletion in oligodendrogliomas) remain stable across treatment, whereas late-stage alterations show dramatic evolutionary divergence. TMZ exposure selects for MMR-deficient clones that subsequently undergo explosive mutagenesis, often accompanied by driver mutations in growth factor signaling pathways (PDGFRA, MET, EGFR) and cell cycle regulators (CDKN2A/B, RB1) [31,32] (Figure 2). CDKN2A homozygous deletion was the most frequently acquired alteration after chemoradiation, present in grade 4 tumors but often absent in the primary lower-grade disease [32]. Concomitant acquired activating PDGFRA or MET alterations frequently co-occur with CDKN2A loss, suggesting coordinated selection for proliferative advantage and the evasion of senescence during malignant progression. Importantly, these recurrence-associated alterations represent clonal selection under therapeutic pressure rather than direct TMZ-induced mutagenesis. At initial diagnosis, CDKN2A/B homozygous deletion is present only in approximately 10% of IDH-mutant gliomas, but increases to 30% at recurrence following treatment [31,33]. Similarly, PDGFRA amplification and RB1 inactivation are rare at diagnosis but emerge frequently at recurrence. Rautajoki et al. demonstrated that CDKN2A/RB1 inactivation was most common in tumors that received chemoradiation compared to radiation alone, supporting treatment-mediated selection [32].

### 3.2. Spatial and Temporal Heterogeneity

Multi-region sequencing studies demonstrate that TMZ-induced hypermutation drives extraordinary spatial heterogeneity within recurrent gliomas [20,34]. While founding driver mutations remain ubiquitous across sampling sites, the hypermutator phenotype generates thousands of private passenger mutations that differ between geographic regions of the same tumor. This spatial divergence reflects both the stochastic nature of TMZ-induced mutagenesis and differential selection pressures across the tumor microenvironment. Importantly, the “distant recurrence” phenotype, i.e., whereby tumors recur in brain regions remote from the original site, shows a strong association with hypermutation and arises from highly mutated subclones [23,35].

The temporal dynamics of hypermutation also reveal important information. Liquid biopsy studies using cerebrospinal fluid (CSF) cell-free DNA demonstrate that the SBS11 signature can be detected early during recurrence or prior to recurrence detection via radiological assessment [36]. This time interval between molecular and imaging detection creates a potential for adaptive intervention, a concept explored further in Section 6.

### 3.3. Cellular State Plasticity and Glioma Stem Cell Dynamics Under TMZ Pressure

Glioblastoma cellular heterogeneity extends beyond genetic diversity to encompass transcriptional plasticity among distinct cellular states. Single-cell RNA sequencing has revealed four major cellular states: oligodendrocyte precursor cell-like (OPC-like), neural progenitor cell-like (NPC-like), astrocyte-like (AC-like), and mesenchymal-like (MES-like), with tumors dynamically transitioning between these states [37]. Recent single-cell analyses examining glioma cellular composition before and after TMZ treatment have revealed that NPC-like clusters exhibit particularly strong stemness features and an enhanced DNA repair capacity, potentially contributing to treatment resistance [38]. Notably, the acquisition of the MES-like phenotype is associated with treatment failure and disease recurrence, including TMZ treatment [39]. Glioma stem cells (GSCs), marked by the transcription factors SOX2 and OLIG2, among others, promote stemness and TMZ resistance through multiple mechanisms [40]. One mechanism is GSC-mediated senescence escape. While the majority of glioma cells are eradicated by TMZ, a subset enters cell cycle arrest and adopts a senescence-associated secretory phenotype [41]. These cells eventually escape senescence, re-enter the cell cycle, and form aggregates exhibiting enhanced stem-like characteristics including elevated stemness marker expression, increased invasiveness, and chemotherapy resistance.

## 4. Clinical Evidence on TMZ-Induced Mutagenesis

### 4.1. The Immunogenic Enigma: TMB, Neoantigens, and Immunotherapy in Glioblastoma

As presented above, TMZ-induced hypermutation generates the highest TMB observed across adult gliomas, frequently exceeding 100 Mut/Mb—comparable to or higher than melanoma and non-small-cell lung cancer, malignancies exquisitely sensitive to immune checkpoint inhibitors (ICIs) [42]. TMB is a known predictor of benefit from ICIs, yet ICIs have failed in glioblastoma. Three landmark phase III trials (CheckMate143, CheckMate498, CheckMate548) demonstrated no survival benefit from ICIs in glioma [43,44,45]. In CheckMate143, nivolumab (PD-1 blockade) was compared with bevacizumab in patients with recurrent glioblastoma. Notably, it was the only study to recruit patients with prior exposure to TMZ, and no formal TMB-high subgroup analysis was published. The CheckMate498 trial compared ICI vs. TMZ in CheckMate548, while comparing ICI + TMZ + RT vs. TMZ + RT alone, and the treatment was given concurrently and not in a sequential manner. To date, no formal analysis has compared ICI + vs. placebo in TMZ-induced TMB-H gliomas. The phase II NCT02658279 study, evaluating pembrolizumab in patients with recurrent malignant glioma with a hypermutator phenotype, has not yet published results. However, retrospectively, Touat and colleagues showed that 11 patients with TMB-H MMR-deficient glioma who were treated with ICIs showed no benefit compared to 199 TMB-L patients [20].

As TMB serves as a surrogate for neoantigen load in many cancers, this enigma demands mechanistic explanation. Interestingly, a study showed that TMZ-hypermutant gliomas are highly immunogenic and are rejected outside the brain (subcutaneous models), but are not rejected when placed inside the brain (orthotopic in vivo models) [46]. This highlights the brain microenvironment itself as a dominant suppressive force, “stunting” the immune response that the neoantigens should have triggered. Two bold landmark phase II studies explored TMZ-induced hypermutation in immunogenic “cold” tumors outside the CNS. The researchers aimed to explore whether TMZ can sensitize MGMT-silenced, microsatellite stable (MSS) metastatic colorectal cancer (mCRC) to ICIs [47,48]. In the MAYA trial, TMZ priming was followed by low-dose ipilimumab (CTLA4 blockade) plus nivolumab, while in the ARETHUSA trial (NCT03519412), TMZ priming was followed by pembrolizumab only if the priming resulted in TMB ≥ 20 Mut/Mb. Both studies demonstrated the TMZ signature SBS11 in blood ctDNA. Responses were 45% objective response rate (ORR) in MAYA, 0% ORR in ARETHUSA (0/6), but 50% showed stable disease (3/6). Despite these phase II findings, both published in 2021–2022, no phase III trials have been initiated to further evaluate this sequential TMZ-then-ICI approach as of November 2025.

**Figure 3:**
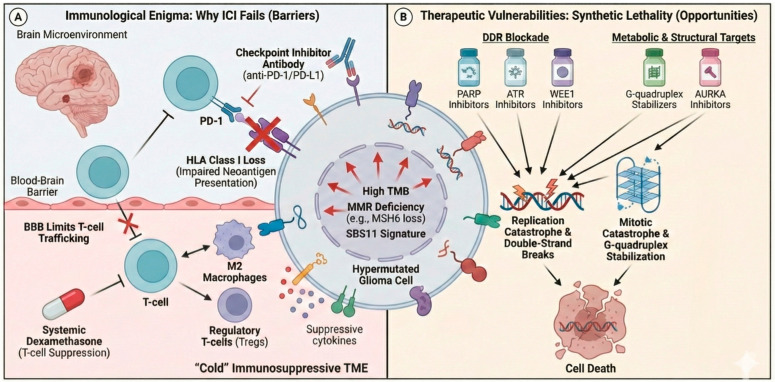
The “immunogenic enigma” and therapeutic vulnerabilities in hypermutated gliomas. (**A**) The immunogenic enigma: despite exhibiting a high tumor mutational burden (TMB) and potential neoantigen load, hypermutated gliomas often fail to respond to immune checkpoint inhibitors (ICIs). This resistance is driven by multiple barriers: the blood–brain barrier (BBB) limits T-cell trafficking; systemic dexamethasone use suppresses immune function; the tumor microenvironment is immunosuppressive (“cold”), dominated by regulatory T-cells (Tregs) and M2 macrophages; and tumor cells may lose HLA Class I expression, impairing neoantigen presentation. (**B**) Therapeutic vulnerabilities: Hypermutation creates specific synthetic lethal opportunities. DDR Blockade: the inhibition of DNA damage response proteins (PARP, ATR, or WEE1) in the context of high-replication stress leads to replication catastrophe and double-strand breaks. Metabolic and structural targets: agents that stabilize G-quadruplex structures or inhibit mitotic regulators like AURKA can drive the cancer cells into mitotic catastrophe and cell death. Google’s Nano Banana Pro assisted in creating the illustration.

Multiple immunosuppressive mechanisms can explain the decoupling of TMB from immune responsiveness [49] (Figure 3A). First, the immunologically “cold” tumor microenvironment (TME). Glioblastoma maintains a profoundly immunosuppressive TME, exhibits profound T-cell exclusion, minimal PD-L1 expression, and the enrichment of immunosuppressive myeloid cells (Myeloid-derived suppressor cells, M2 macrophages) and regulatory T cells [50,51,52]. TMZ itself may exacerbate this through lymphodepleting effects, reducing the pool of tumor-reactive effector cells [51]. Furthermore, glioblastomas rarely develop tertiary lymphoid structures, organized immune hubs present in checkpoint-responsive tumors that sustain antitumor immunity [53,54]. Second, molecular heterogeneity and plasticity: single-cell analyses reveal that glioblastoma comprises multiple transcriptional states, including stem-like, mesenchymal, and differentiated phenotypes [37]. This intratumoral heterogeneity enables rapid adaptation through transcriptional plasticity, potentially allowing tumor cells to evade immune recognition without genetic evolution. TMZ-induced hypermutation might amplify this heterogeneity. Moreover, while TMZ induces mutations and neo-peptides, its subclonal, heterogeneous nature might result in a less immunogenic environment [20,55,56]. Loss of HLA class I expression or mutations in antigen presentation machinery (such as TAP1/2 and β_2_-microglobulin) represent a widespread immune escape mechanism in various cancers, including glioblastoma [57,58]. The loss of HLA prevents neoantigen presentation to CD8^+^ T cells, irrespective of the mutational burden, thereby decoupling TMB from immune recognition. Lastly, most glioblastoma patients are treated with dexamethasone to manage peritumoral edema. A subgroup analysis of the CheckMate143 trial revealed that baseline use of dexamethasone was associated with significantly worse survival among nivolumab-treated patients compared to those receiving bevacizumab [43]. Preclinical studies in glioblastoma models demonstrate that dexamethasone abolishes ICI efficacy in a dose-dependent manner by suppressing T-cell proliferation, trafficking, and effector functions [59].

### 4.2. Acquired Resistance Mechanisms

Beyond MMR deficiency as an escape mechanism [60], several other mechanisms contribute to acquired resistance to TMZ. Long non-coding RNA (lncRNA) networks can undergo remodeling in TMZ-resistant gliomas [22,61,62,63]. Expression profiling of IDH-mutant low-grade gliomas treated with TMZ identified a three-lncRNA signature (including HOXD-AS2 and H19-related lncRNAs) that is correlated with TMZ resistance and progression-free survival [22,62,64]. Functional annotation suggested that these lncRNAs are involved in cell proliferation and differentiation through interaction with cancer-related genes like SMAD2 and UBR5 [62]. The mechanistic basis involves chromatin remodeling and transcriptional reprogramming, which enable cells to evade TMZ-induced damage. Genomic analyses reveal frequent acquisition of activating alterations in PI3K/AKT/mTOR and receptor tyrosine kinase (RTK) pathways in recurrent disease [32,65]. PDGFRA amplification, MET mutations, and EGFR alterations emerge under therapeutic pressure, often in conjunction with CDKN2A loss [20,31,32,66]. These alterations restore proliferative capacity and may confer survival advantages independent of TMZ sensitivity. Targeting these pathways represents a rational combination strategy; however, clinical trials of PI3K or mTOR inhibitors as single agents have not demonstrated a clinical benefit [67,68]. Post-translational protein modifications can also affect resistance to TMZ. Histone lactylation (H3K9la) reduces MLH1 expression in recurrent glioblastoma, weakening MMR and contributing to TMZ resistance [69]. TRIM25 expression is frequently upregulated in glioblastoma, correlating with higher tumor grades and resistance to TMZ. Elevated TRIM25 levels are associated with poor prognosis and enhanced tumor growth both in vitro and in vivo. Mechanistically, TRIM25 inhibits oxidative stress and ferroptotic cell death during TMZ treatment by promoting the nuclear import of Nrf2 through Keap1 ubiquitination, highlighting its role in glioblastoma chemoresistance and its promise as a therapeutic target [70]. Exosome-transmitted circCABIN1 represents emerging evidence that the exosome-mediated transfer of circular RNAs, such as circCABIN1, plays a crucial role in promoting TMZ resistance in gliomas by sustaining downstream signaling and enhancing cancer stemness features. The dissemination of drug resistance via exosomal circRNAs highlights a novel intercellular communication mechanism and suggests promising therapeutic strategies for overcoming acquired chemoresistance. Advances in engineered exosome delivery systems may further enable targeted interventions aimed at restoring TMZ sensitivity and improving patient outcomes [71]. Lastly, global transcriptomic analyses have identified HMOX1, LTF, and STEAP3 as iron metabolism-related genes (IMRGs), which are strongly associated with mesenchymal transformation, TMZ resistance, and poor prognosis [72]. The knockdown of these genes reduces glioma cell proliferation, migration, and invasion [72]. The iron metabolism–TMZ resistance axis remains mechanistically incompletely understood.

## 5. Therapeutic Opportunities and Strategies

### 5.1. Targeting the DNA Damage Response

The DNA damage response (DDR) dependency created by TMZ-induced genomic instability presents exploitable vulnerabilities. Hypermutated, MMR-deficient gliomas accumulate replication stress, rely on alternative repair pathways, and display synthetic lethality with DDR inhibitors. Poly(ADP-ribose) polymerase (PARP) inhibitors target defects in homologous recombination repair and base excision repair. While earlier-generation PARP inhibitors (e.g., olaparib, talazoparib) showed lower brain penetration, among others, due to the blood–brain barrier and efflux transporter activity, newer agents such as niraparib achieved higher CNS concentrations [73,74,75]. In a preclinical study using patient-derived xenograft cell lines, niraparib combined with TMZ increased the expression of NKG2D ligands (ULBP1) on glioblastoma cells in vitro, enhancing γδ T-cell-mediated cytotoxicity in co-culture assays. Veliparib, another brain-penetrant PARP inhibitor, demonstrated TMZ sensitization in patient-derived xenograft (PDX) models, though clinical translation has been limited by overlapping hematologic toxicities with TMZ [75,76]. As for ATR inhibitors, ataxia telangiectasia and Rad3-related kinase (ATR) regulate replication, stress responses, and cell cycle checkpoints. Preclinical studies demonstrate that ATR inhibition induces synthetic lethality in MMR-deficient gliomas [77,78,79,80]. In 2024–2025, three selective ATR inhibitors advanced in development (Camonsertib, phase II; AD1058, preclinical; YY2201, preclinical), of which the preclinical AD1058 was shown to be a good brain-penetrator [81,82,83]. Regarding the WEE1 and CHK1 inhibitors, WEE1 kinase and checkpoint kinase 1 (CHK1) regulate G2/M cell cycle transition and DNA damage checkpoints, downstream to ATR, and have also emerged as promising therapeutic targets, as shown in several recent studies [84,85,86].

### 5.2. Reimagining Immunotherapy for Hypermutated Gliomas

The failure of ICI monotherapy does not preclude immunotherapy success; rather, it demands more sophisticated approaches. First, while there are multiple studies showing non-impressive responses to PD1 inhibitors in glioblastoma, small-sized retrospective evidence for TMZ-induced hypermutation tumors, and preclinical mechanisms for these findings, it is important to point out that there are no prospective studies or subgroup analyses published explicitly for TMZ-induced hypermutated phenotypes in gliomas. Moreover, given the subclonal nature and reduced antigen presentation, a mutational load response might be beneficial. Perhaps ultra-hypermutators would benefit from ICIs, while the currently used cutoffs of 10–30 Mut/Mb are not enough in the case of gliomas. A 2025 proof-of-concept case report described the neoadjuvant administration of a triplet immune checkpoint blockade (nivolumab, ipilimumab, and relatlimab targeting PD-1, CTLA-4, and LAG-3, respectively) in a single patient with newly diagnosed IDH-wildtype, MGMT-unmethylated glioblastoma [87]. Following a single dose administered 12 days before resection, marked tumor-infiltrating lymphocyte infiltration and activation were observed compared to the baseline biopsy, with no evidence of recurrence at 17 months. A phase III clinical trial (GIANT; NCT06816927) is planned to prospectively evaluate this strategy in a larger cohort. Therefore, the PD1-CTLA4-LAG3 triplet in TMZ-induced TMB-H would also be of interest. Lastly, while multiple advanced immunotherapies are being investigated in gliomas, these are beyond the scope of our TMZ-centered review.

### 5.3. Exploiting Acquired Vulnerabilities

Lomustine (CCNU) may show activity against MMR-deficient gliomas that are TMZ-resistant [88,89]. The drug, from the nitrosourea (NU) class, generates distinct DNA lesions, including interstrand crosslinks not requiring MMR for cytotoxicity. Another NU drug, BCNU, has demonstrated in vitro activity against glioblastoma cell lines, and a carmustine wafer (Gliadel) is FDA-approved for the intratumoral treatment of glioblastoma. Recently, this class has gained clinical attention, as reviewed in [90]. A landmark preclinical study demonstrated that the dual inhibition of the EGFR/AKT and mevalonate pathways synergistically enhances the antitumor activity of TMZ in glioblastomas by inducing metabolic reprogramming and exposing vulnerabilities in energy metabolism. Mechanistically, this combination remodels tumor-cell lipid metabolism and downregulates fatty acid synthesis genes in an NF-κB-dependent manner, highlighting a promising therapeutic strategy for EGFR-overexpressed or mutated glioblastoma [91]. Alternative splicing has also been gaining attention [92,93]. TMZ resistance has been shown to create vulnerabilities in the form of guanine mutations that disrupt G-quadruplex DNA structures and G-rich RNA splice sites. Tiek et al. demonstrated that TMZ-resistant gliomas become selectively sensitive to G4-stabilizing agents and splicing kinase inhibitors [93]. TMZ-induced hypermutation frequently co-occurs with chromosomal instability and dysregulated mitotic control. A 20-gene CDC20-associated mitotic signature (CDC20-M), comprising genes controlling chromosome segregation and mitotic checkpoints identifies gliomas with high genomic instability and TMZ resistance [94]. The CDC20-M signature was validated as an independent predictor of poor survival across more than 1000 glioma patients in four independent datasets, with high-risk tumors exhibiting significantly shorter progression-free and overall survival [94]. Aurora kinase A (AURKA), a master regulator of mitotic spindle assembly and chromosome segregation, emerged as a tractable therapeutic target within this signature. Subsequently, the AURKA inhibitor MLN8237 (alisertib) demonstrated efficacy in preclinical glioma models [95]. Early-phase clinical work has combined alisertib with radiation in high-grade gliomas with acceptable toxicity, and preclinical data support synergy with TMZ and radiation [95,96] (Figure 3).

## 6. Real-Time Monitoring with Liquid Biopsy from CSF and Plasma

Liquid biopsies using cell-free DNA (cfDNA) or circulating-tumor DNA (ctDNA) from plasma or from CSF represents a transformative advance for the real-time monitoring of glioma evolution [36,97,98]. The latest advancements have allowed for the detection of mutational signatures with low numbers of mutations, such as those derived from targeted gene panels [99,100]. Moreover, unlike plasma, where circulating-tumor DNA (ctDNA) is sparse due to blood–brain barrier restrictions, CSF yields robust ctDNA signals [36]. Therefore, CSF might serve as a superior reservoir compared to plasma for ctDNA profiling in gliomas, offering a reliable modality for longitudinal monitoring and the detection of genomic evolution, as supported by several 2025 publications [98,101]. Prospective genomic analysis demonstrates a ctDNA detection rate of approximately 50–60% in CSF, with high concordance (84%) with the primary tumor and longitudinal CSF sampling-captured temporal evolution, including specific utility in monitoring treatment resistance, capable of detecting the specific hypermutation signatures associated with acquired resistance to TMZ therapy [98].

## 7. Future Directions and Conclusions

Altogether, the TMZ era in neuro-oncology has revealed a fundamental effect: effective treatment that inevitably drives tumor evolution. This duality of TMZ as both a life-prolonging therapy and a potent evolutionary driver necessitates careful consideration of the risk–benefit balance, with two unanswered questions: who are the patients that may develop the hypermutator phenotype? How can TMZ be exploited as an induction therapy to sensitize tumors to successful treatments? We propose three interconnected research avenues with promising transformative implications: (i) integrating spatial transcriptomics, proteomics, and epigenomics to map how TMZ-induced mutations rewire cellular states and tumor–immune interactions at single-cell resolution. Understanding why certain hypermutated regions remain immune-excluded while others permit infiltration may unlock combination immunotherapy strategies. (ii) AI-driven neoantigen prediction with machine learning algorithms that integrate, among others, mutational context, HLA typing, proteasomal processing predictions, and TCR repertoire profiling, could identify the rare immunogenic neoantigens within thousands of TMZ-induced passengers. Targeting these computationally prioritized neoantigens through vaccines or TCR-engineered T cells may overcome the quantity–quality enigma. (iii) Advanced organoid models such as patient-derived organoids that recapitulate tumor heterogeneity, MMR status, and microenvironmental features, enabling the functional testing of evolutionary dynamics and therapeutic combinations ex vivo. Organoid drug screening could identify patient-specific vulnerabilities before clinical trial enrollment, personalizing therapy selection.

In conclusion, the synthesis presented here repositions TMZ from a first-line cytotoxic agent to a central driver of glioma evolution, whose variable mutagenic legacies shape subsequent therapeutic decisions. Recognizing hypermutation as both a challenge and an opportunity opens new avenues toward precise and evolution-aware brain tumor management. Future, carefully designed clinical trials should elucidate the clinical relevance of the translational findings and ultimately pave the way to improved patient outcomes.

## Data Availability

No new data were created or analyzed in this study.
